# Dental Education: A Guide for Novice Tutors

**DOI:** 10.7759/cureus.43227

**Published:** 2023-08-09

**Authors:** Muslat A Bin Rubaia’an

**Affiliations:** 1 College of Medicine, King Saud University, Riyadh, SAU; 2 College of Dentistry, Riyadh Elm University, Riyadh, SAU

**Keywords:** studying, bachelor, teaching, student, teacher, undergraduate, learning

## Abstract

The primary objective of undergraduate-level dental education is to produce proficient dental practitioners who can effectively address the oral health needs of the community and enhance the overall oral health of the population. The field of dental education is subject to continuous change that is shaped by many factors, including changing societal norms, shifting responsibilities of dental practitioners, changing healthcare environments, and rapidly evolving dental science. Learning theories significantly impact the advancement of dental education, and educators must recognize and acknowledge their influence. Dental faculties must be adequately prepared and motivated to use innovations, which enable them to impart knowledge in a practical and organized manner. This review provides an overview of teaching methodologies that have gained acceptance in recent years. It highlights the importance of their implementation in facilitating an effective teaching and learning process in consideration of their history, style, and core focus. A clearer understanding of these techniques can enhance education standards, help establish dental instructors’ responsibilities and career advancement, and provide insights for future research.

## Introduction and background

The history of dental education is characterized by continuity and change [1]. As dental science and technology have advanced, the content and methodology of education for dental practice have become more sophisticated. A lengthy period of formal instruction has replaced apprenticeship training and purely self-taught and self-proclaimed competence. Independent for-profit schools have given way to university-based schools of dentistry, and postgraduate education in general and specialized areas of practice has become ubiquitous [1].

Dental education is a component of medical education and the medical field. Charles H. Mayo, MD, addressed the American Dental Association in 1928, stating: “The practice of medicine includes dentistry, and dentistry is the practice of a special branch of medicine” [2]. Medical education is an art with scientific principles and a form of learning involving the generational transfer of knowledge, skills, and attitudes through teaching, training, research, and practice [3]. The process of education should enable modifications in the cognitive, affective, and behavioral domains of the learner while also imparting fundamental knowledge and competencies in a specific scientific discipline [3].

Medical education is distinctive from many other fields of science, technology, literature, and art because it focuses on human life and well-being and because of the specific knowledge, skills, and conduct that it requires [3]. In a lecture at the State of Maryland in 1913, medical historian Eugene Cordell criticized the lack of official teaching on the history of medicine [4]. He emphasized the need for this information throughout all educational program levels. A century later, in support of Dr. Cordell’s presidential speech, Yarnall stressed the necessity of studying the history of medicine to learn from the achievements and errors [4,5].

The documented history of medical practice and medical education dates back to ancient China, Babylon, Egypt, and India [6]. During the Middle Ages, formal acknowledgment of medicine increased across Europe [6]. Medicine was performed as an apprenticeship program in monasteries under the control of hospitals in the 1220s. In 1310, Dr. Gaddesden wrote the earliest surviving British medical textbook, Rosa Anglica [4,7]. In 1421, the British Parliament campaigned for the passage of legislation restricting medical practice to those with university-granted qualifications [8]. Years later, few medical professionals considered dentistry appealing or challenging [1]. Various types of surgical procedures were also perceived as unexciting and mechanical. However, oral health problems and treatments, in particular, became increasingly isolated from the field of medicine [1]. In the 16th century, significant progress in anatomy, microbiology, and related disciplines established the groundwork for developing specialized writings on dentistry [1]. Moreover, this period witnessed a shift toward a theoretical perspective in oral health, moving beyond mere mechanical and empirical approaches [1]. The aforementioned advancements encompassed comprehensive anatomical elaboration of teeth and associated structures. In 1728, during a period when dentistry had achieved greater professionalization, Fauchard, frequently credited as the father of contemporary dentistry, authored a two-volume publication on the field of dentistry [9]. This work represented the initial comprehensive explanation of the subject. Fauchard recognized the necessity for schools of surgery that would offer dental education, but he also recognized the lack of written resources that could be used to guide such learning [1]. The year 1840 marked the establishment of the Baltimore College of Dental Surgery, the first dental college [9]. This development highlighted the necessity for increased regulatory supervision in the field.

From that point forward, the notion of dental education has evolved into two branches: odontology and stomatology [10]. Gradually, odontology lost its relationship to medicine and general health. Additionally, stomatology did not highlight dental competencies and procedures. The educational system has recently shifted toward odontology due to the Bologna process, and many problems relating to these two distinct features have been resolved [10].

Undergraduate dentistry education focuses on creating competent general dentists who are capable of examining, diagnosing, treating, and managing diseases and disorders in the oral region of the human body, leading to a Bachelor of Dental Surgery (BDS) degree. In particular, dental education provides the general dentist with significant biochemical and clinical subject understanding. Dentists must also have good interpersonal communication and social skills, utilize technology successfully, demonstrate professional behavior guided by an ethical code, and exhibit critical thinking and problem-solving skills. The abilities required during dental education are known as dental competencies, and their mastery is proven when practicing independently without supervision and without causing injury to a patient. These competencies include comprehending situations, displaying critical-thinking ability, competence, self-directed learning proficiency, problem-solving skills, moral ideals, and practical and mechanical capabilities [11,12].

Education is a dynamic process that requires regular refinement [13]. However, academic medical curricula currently do not exploit innovative teaching techniques, making them inadequate for making substantial advancements in the future [13]. The efficacy of undergraduate dental education is crucial for preparing students for their future careers. To improve student learning, participation, and motivation, educators must develop various strategies for enhancing the educational experience. Understanding the different learning styles of students and adapting pedagogical strategies to accommodate these styles are vital for improving student performance. Thus, this review aims to describe and evaluate various forms of teaching and learning that can be used in addition to or as a replacement for traditional lectures to aid in active learner engagement.

## Review

Learning approaches

Students may learn by understanding, rote memorization, and reproduction of memorized information or through various degrees of combinations of these strategies [14]. Learning approaches constitute cognitive, affective, and psychosocial behaviors that are relatively stable indicators of how learners perceive, interact with, and respond to the learning environment. They involve instructional strategies that allow individuals to acquire knowledge most efficiently [15].

In general, two concepts are frequently utilized in educational research on approaches to learning: the “surface approach” and the “deep approach” [14]. When attempting to reproduce course material, students who have utilized a surface approach focus on memorization and rote learning of the text. Conversely, those who use a deep approach seek to comprehend the text’s intent, value, and meaning. Depending on the requirements of the context, the strategic approach may be incorporated into either deep or surface processing [16]. Students may employ either deep or surface strategies or a combination of them throughout their studies. Significant roles are played by course design, assessment design, and instructional methods in fostering deep, surface, and strategic learning [14].

Surface Approach

In the surface approach to learning, students concentrate on details and sections of information that are deemed essential. Individual components or pieces of information must be memorized in order to demonstrate sufficient comprehension to complete the assignment. In a superficial learning strategy, tasks are viewed as burdens or obstacles to be overcome. The focus of surface learning is, “What do I require in order to pass?” Knowledge may need to be more superficial and effective in fostering comprehension. Learners may concentrate on unrelated facts that they must reproduce later on in examinations or in other assessments.

One trait of the surface method is a propensity to “lose the point” of learning. Failure aversion is also believed to be the primary motivation for surface learning. Surface approaches are driven by the learner’s inclination to fulfill the basic criteria with limited exertion and involvement, leading to unsatisfactory educational achievement [17-19].

Deep Approach

A deep approach is a structured approach that emphasizes comprehending concepts and establishing connections between ideas [20]. It requires paying attention to the underlying significance and is associated with analytical abilities, cross-referencing, creative reconstruction, and independent thought [21]. Deep learning fosters critical thinking and promotes the long-term retention of concepts. In this approach, students examine the significance of what is being taught and attempt to make sense of it by connecting information and thought to the topic. They seek the overall meaning and attempt the holistic processing of information.

Students construct their own interpretations of the material by incorporating it with prior knowledge. Consequently, deep learning is regarded as the preferred learning style in higher education [20]. Students who employ a deep approach to learning are distinguished by their focus on the task at hand and their inherent drive to participate actively in the studied material. They strive to comprehend the material fully and derive personal fulfillment from their studies. Systematic execution of these tasks typically leads to profound comprehension [22,23].

To facilitate an active learning process, students engage in the monitoring and restructuring of their cognitive processes as they engage with course materials. This involves critical analysis of the material in relation to other experiences and ideas, as well as the integration of formal knowledge with personal experience. Additionally, students are expected to establish connections between factual information and their corresponding conclusions [17,19,22,24].

Strategic Approach

Another approach that learners often take is the strategic approach. Some students may use deep and surface approaches to achieve their objectives according to what is required and the conditions under which they are learning, such as the time available to prepare for an examination. This practice is known as strategic learning [25]. Strategic learners use “cues and clues” related to topic assessments and are driven by learning that leads to positive outcomes, such as excellent grades [26]. This type of learning is characterized by heightened awareness of assessment and monitoring, resulting in a dispersed understanding of the subject matter and poor integration between topics [24].

The strategic approach involves the learner’s motivation to maximize performance to excel and achieve the highest possible grades through organized study skills and prudent time management [26,27]. Moreover, assessment task requirements heavily influence students’ study habits but are generally highly structured and productive [18,25,28]. This approach is also characterized by competitiveness and attempts to maximize academic success with minimal effort [28]. Characteristics of the types of learners discussed thus far are summarized in Table 1 [29].

**Table 1:** Characteristics of different types of learners.

Deep learner	Surface learner	Strategic learner
Actively attempt to comprehend the material/subject	Learn in order to replicate what has been learned	Intend to achieve excellent grades
Engage actively with the content	Memorize information required for exams	Schedule time and allocate efforts for maximum impact
Utilize evidence, investigation, and evaluation	Take a narrow perspective and focus on specifics	Ensure that the study environment and content are suitable
Relate new ideas to prior understanding	Failing to differentiate principles from instances	Utilize question banks from previous exams to anticipate questions
Read and study beyond what is required for the course	Typically adhere tightly to course specifications	Utilize grading criteria with care
Motivated by passion/interest	Motivated by failure anxiety	Motivated by grades

Adult learning theory

Andragogy-based learning theories guide all higher-education programs, including programs for healthcare professionals [6]. Andragogy emphasizes the consideration of “adult learners” as participants in the teaching-learning process who can determine their own learning needs based on various experiential backgrounds, abilities, and motivations. The task of conveying a substantial quantity of information within a restricted timeframe in a manner that is comprehended, retained, and meaningfully interpreted by a learner is a significant challenge. Hence, traditional approaches, such as teacher-based approaches, have been challenged substantially by the new adult theory [30].

**Figure 1:**
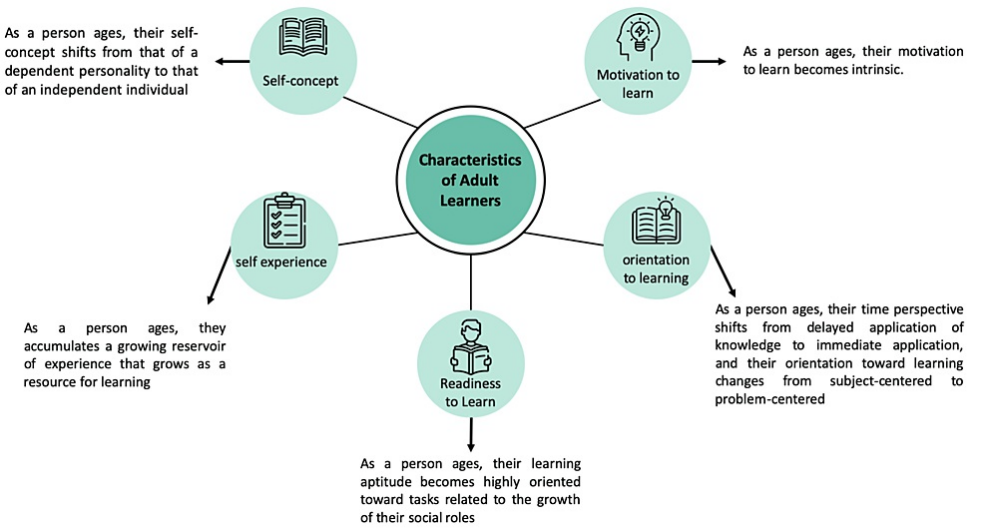
Malcolm Knowles’ five assumptions about adult learners. The author designed the figure (icons: Flaticon.com). The content was cited from eLearning Infographics [34].

Educational programs aimed at healthcare professionals are commonly based on diverse philosophical and theoretical frameworks that offer direction in designing and developing curricula for educational courses [4]. Several adult learning theories are based on a constructivist philosophy that requires building new knowledge on existing information. This is consistent with the beliefs offered in andragogy, which Alexander Kapp developed in 1833 [31]. In 1980, Knowles established four assumptions about the characteristics of adult learners (andragogy) that contrasted with his assumptions regarding child learners [32]. Knowles added a fifth assumption in 1984 (Figure 1) [33,34].

The literature classifies adult learning theories as instrumental, humanistic, transformative, social, motivational, reflective, and constructivist [6]. These theories are developed from psychological theories of learning. They were inspired by the wide constructivist perspectives of andragogy, which indicate that learning generates new information based on current knowledge [35]. These theories are described below.

Instrumental learning theories

Instrumental learning theories comprise behavioral theories, cognitivism, and experiential learning.

Behavioral Theories

Behavioral theories are a consequence of focusing on a stimulus in the environment that results in a modification of an individual’s behavior [36]. Positive outcomes, or reinforcers, improve behavior and learning, whereas adverse outcomes, or sanctions, undermine it [35]. According to the behaviorist paradigm, educators bear the responsibility of controlling the learning environment to elicit the intended response, thereby demonstrating a teacher-centric approach to education.

Cognitivism

Instead of focusing on the external context or environment, the emphasis in cognitivism is on the learner’s internal environment and its cognitive structures [37]. Cognitive learning theories pertain to the mental and psychological mechanisms that facilitate learning by attributing significance to various experiences, which include perception, information processing, reflection, metacognition, and memory, among others. Knowledge acquisition primarily occurs within the confines of structured educational settings, where instruction is delivered through oral or written means and through demonstrations and involves the explicit and recognizable accumulation of knowledge.

Experiential Learning

In experiential learning, the process of acquiring knowledge and constructing new understanding is enhanced by active involvement with genuine surroundings [36]. According to Kolb, experience facilitates learning and knowledge formation, and the learning cycle is divided into four phases: concrete experience, reflecting observation, abstract conceptualization, and active experimentation [38]. The experiential learning cycle proposed by Kolb permits apprehension, comprehension, intention, and extension.

Humanistic theories (facilitative learning theories, self-directed learning)

Humanism is a paradigm from the 1960s that emphasizes individual freedom and dignity to realize one’s greatest potential. It presupposes that the learning process is intrinsically motivated and that individuals of mature age possess the capacity to strategize, manage, and assess their cognitive development to attain self-realization, personal satisfaction, self-driven incentives, objectives, and autonomy in their educational pursuits. Thus, the process of acquiring knowledge can be oriented toward the student and customized, with educators serving as facilitators of learning [35].

Transformative learning theories (reflective learning)

Transformative learning theories concentrate on the alteration of meaning, context, and enduring assertions. Mezirow refers to these embedded assumptions as “frames of reference” [39]. Mezirow’s approach enables learners to identify and question the soundness of their ingrained assumptions [36]. Learning occurs when novel information is integrated with pre-existing knowledge, and learners maintain their initial “frame of reference” while also critically evaluating and modifying aspects of their conceptual frameworks [36,40]. The first stage of transformative learning entails encountering a confusing problem and engaging in introspection about prior viewpoints concerning the matter. The second is self-reflection and critical analysis of the experience, which require metacognitive thought. The third stage involves taking action on the issue, which leads to modifications in the significance, situation, and long-established concepts determined by self-reflection and previous beliefs.

Social theories of learning (zone of proximal development, situated cognition, communities of practice)

Social learning theories combine the concept of behavior modeling with cognitive learning to enhance the comprehension of how to complete a task. Social learning theories emphasize social contact, the individual, the environment, the community, and the intended behavior as the primary learning facilitators. Observation and modeling are the core components of social learning theories. Educators are responsible for creating a conducive atmosphere for learning and delineating expected conduct [35,41].

Motivational model (self-determination theory, expectancy valence theory, chain-of-response model)

The motivational model is based on the premise that adult learning involves two key components: motivation and reflection. Self-determination theory focuses on intrinsic motivation, while expectancy valence theory incorporates the expectation of success. The chain-of-response model focuses on three internal motivating factors: self-evaluation, the learner’s attitude toward education, and the significance of goals and expectations [36,42].

Reflective models (reflection on action and reflection in action)

The process of reflection facilitates the comprehension of intricate occurrences and enables students to derive knowledge from their personal encounters [36]. According to Schon, there are two types of reflection: reflection on action and reflection during the action [43]. Reflection on action permits learners to evaluate the level of significance or rigorousness of the processes post-occurrence, whereas reflection during action empowers learners to engage in introspection during the course of their task [44]. This encourages students to investigate their understanding.

The nature of reflective learning is contingent upon the ability of students to engage in introspection about their experiences, clinical challenges, and contextual factors that shape their practice. Given an environment that fosters learning and instructors who provide positive reinforcement, a student’s ability to engage in introspection and practical application can gradually progress. Educators must provide students with a structured reflection guide and helpful feedback on their reflections.

It is important to note similarities in the critical reflection model proposed by Mezirow, as discussed in the context of transformative learning theories, and Schon’s reflection models on action. They both involve introspection on prior assumptions and knowledge, necessitating action to effect change [45-47]. In the literature, “reflection” and “critical reflection” are used interchangeably, but not all instances of reflection are critical. By linking old and new information to assess learning environments holistically, critical reflection generates transformative learning for learners and educators by engaging in higher and more demanding levels of thought [48].

Constructivism (cognitive constructivists and sociocultural constructivism)

The theory of constructivism pertains to both epistemology and psychology. It elucidates the mechanisms involved in the acquisition of knowledge and the development of meaning. Sociocultural constructivism is a social theory of learning that emphasizes the sociohistorical and context-dependent nature of learning and development, which was pioneered by Ausubel and Robinson, Piaget and Cook, and Vygotsky [49-51]. According to constructivist theory, the process of knowledge acquisition is facilitated through the interaction of an individual’s pre-existing knowledge and skills, the knowledge and skills acquired through social interactions with peers and educators, and participation in social events [36]. The relativity of knowledge stems from its active generation, which is contingent upon both the physical and social environment of the learner [52]. The constructivist theory of pedagogy and learning emphasizes the internal cognitive mechanisms that underlie learning, participation, and social interaction.

Learning approaches

In the pre-clinical years of education, lectures have traditionally been the focal point. The didactic lecture has traditionally served as the primary means of imparting knowledge from teacher to learner. Nevertheless, this methodology has encountered several obstacles, which have motivated the adoption of contemporary pedagogical strategies. According to White et al., the design and execution of didactic lectures as an active learning exercise for students are inadequate, resulting in a passive learning experience that fails to enhance learning through collaboration effectively [53]. There is a growing trend among students to exhibit reluctance toward participating in educational activities beyond the confines of the classroom.

Taylor believes that “self-investment” and whole-person goal attainment must be fostered as prerequisites for effective learning through participation in communicative tasks [54]. According to Taylor, such an approach emphasizes the necessity to maintain a non-authoritarian presence throughout this process so that students can feel safe and non-defensive. This allows them to learn not because the instructor requires it, but because they need to achieve their objective. Historiographically, the humanistic method can be viewed as a revival of student-centered thinking, which posed a significant challenge to the old teacher-centered teaching model. In recent years, medical education has undergone effective modifications. More and more teachers have adopted a learner-centered strategy for classroom performance. Teachers’ and students’ participation in teaching and learning disrupts standard teaching patterns. Consequently, this learner-centered strategy can facilitate efficient learning in a relaxed environment.

The acceptance of this style of learning may provide a challenge, particularly for young students who have been educated using teacher-centered methods for 12 years before enrolling in university. Students who greatly appreciate “teacher-focused” approaches or have experienced them may reject the learner-centered approach as frightening, embarrassing, overly intrusive, and exhausting [55]. However, even during the same course, students’ perspectives on learning have been observed to vary, and as they age and grow, so do their perspectives on teaching and learning. According to the literature, students become more accepting and agreeable with each passing year [56]. This idea addresses the main issue of the present study. It is vital and beneficial for students to make progressive adjustments in their learning approach and to adapt to new methods without stress or resistance. However, additional teaching strategies should be implemented to achieve successful learning, and a teacher-centered approach should be avoided.

Dentistry has characteristics that necessitate distinctive educational approaches and development. Teaching and learning in dentistry require that students interact with instructors, particular materials, processes, and patients. Clinical dentistry is characterized by the gradual development and internalization of tacit knowledge as students are incorporated into the profession. Moreover, in medical education programs worldwide, the employment of many learning theories, each with advantages and disadvantages, has resulted in the adoption of various instructional strategies by faculty and varying degrees of attainment of learning outcomes. Bhat et al., Challa et al., and other authors have discussed various approaches, which are summarized below [13,57].

Problem‑based learning

Problem‑based learning (PBL) is an instructional approach in which a problem triggers active learning. PBL is linked to constructivism theory, which depends on the student’s ability to identify and define a problem and recognize the essential learning issues for developing a comprehensive understanding of it [58]. This strategy is based on the collaboration of small groups of students with facilitators to achieve learning objectives [59].

PBL has been found to be efficacious in the classroom. It involves the use of problems to stimulate students’ motivation to identify and apply research concepts, relate gathered information, collaborate effectively, and communicate efficiently [59]. The primary goal of PBL is to improve adult learning skills by engaging students in self-direction and problem-solving, thereby facilitating information retention [60-62]. A fundamental requirement for a PBL curriculum is that “the problem always has to occur first.” This may be designated as one of the program’s guiding principles and has significant implications for the curriculum’s structure [63].

The curriculum is primarily based on problems rather than disciplines, emphasizing integrated learning rather than separation into basic and clinical sciences. Next, the process of acquiring knowledge is enhanced by various factors, including small-group instruction, a student-centered approach, efficient study techniques, and self-directed learning [64]. The curriculum is also determined by outcomes, such as enhanced practical knowledge, skill development, the motivation required for continuous learning, and the growth of self-evaluation techniques [64].

Compared to traditional methods, the benefits of PBL include greater assimilation of basic and clinical techniques; significantly refined communication, teamwork, and independent learning; and a greater propensity to work together on a problem, as well as greater enjoyment of such collaboration [57]. PBL teaching has been utilized in medical education for over four decades [65]. The Faculty of Odontology in Malma, Sweden, was the first to implement PBL in dental education in 1990 [57]. From that point forward, PBL has been implemented in dental schools across the globe, and the implementation of this novel idea has been expanding.

PBL students have superior problem-solving skills and attitudes toward research compared to conventional students. In addition, after graduation, PBL students demonstrate significant improvements in preventive care and diagnostic performance. The effects of the PBL methodology have been documented. PBL has demonstrated efficacy in augmenting various competencies in students, including critical and interdisciplinary thinking, patient communication, collaborative aptitude, problem-solving acumen, and self-directed work proficiency [59].

In addition, research has demonstrated that 78% of students found PBL sessions engaging, and 52.3% found it beneficial for enhancing their knowledge, skills, and critical thinking abilities [59,66,67]. Students in another study reported that their knowledge was more memorable and easily recalled during PBL sessions compared to other instructional methods [68]. Notably, student interest in PBL sessions varies across years, with a gradual increase occurring beginning in the first year (29.7%) and culmination occurring in the final year (70.0%).

Although PBL teaching has been widely utilized, its application in American medical colleges has decreased, primarily due to the time-consuming and labor-intensive nature of training for PBL. Therefore, it has been questioned whether it is feasible to devote additional personnel to PBL training [65]. Some students have stated negative opinions about PBL, pointing to the fact that some students dominate the discussion, whereas others remain passive [59]. This can be linked to the diverse behavioral performance and learning styles of students in PBL classes. Azer et al. examined the variables that may influence group interactions, such as students’ and tutors’ perceptions, backgrounds, group dynamics, training, and the nature of the problem being solved [69]. The poor participation of some students during PBL class could be attributed to several factors, such as their prior knowledge of the scenario’s content, their English proficiency, the facilitator’s failure to ensure the involvement of all students, and poor communication among group members [59,70].

To achieve the intended results, educational planners should ensure that PBL groups contain sufficient students with different learning styles, students should be informed about their learning styles, and they should learn self-study methods that compensate for any deficiencies in PBL sessions [71]. Periodic assessment of student performance during PBL sessions can be complex, necessitating the establishment of definitive evaluation techniques. Students should have concurrent access to equivalent resources to benefit from the same advantages as their peers. Even with information overload, the practical completion of case objectives can be improved. In addition, implementing PBL requires students to make a concerted effort to assume responsibility for achieving higher exam scores [13].

The success of PBL relies upon student and instructor collaboration. Students must be adequately prepared for class, allowing them to clear up any misunderstandings or knowledge gaps [13]. Students should also be encouraged to conduct peer evaluations to promote peer learning. In addition, productive PBL employment is possible with an interprofessional approach centered on teamwork, collaboration, and assured professional identity [72].

Case-based learning

Case-based learning (CBL) is an instructional strategy that is characterized by interaction and student-centeredness and is facilitated by an instructor. The methodology promotes active learning using clinical case scenarios that closely resemble real-life experiences that medical students will encounter during the clinical phase of their education [57]. This pedagogical approach is based on constructivism theory and was first developed at Harvard Medical School during the 1920s. Since then, it has been recognized as a successful educational model for health professionals, including those in the field of dental education [73,74].

CBL has been implemented to foster active learning in response to the waning motivation observed in traditional didactic lectures [75]. Students are allowed to investigate authentic cases that provide patient history, signs, symptoms, and clinical and laboratory findings [13]. Using collaborative efforts and peer engagement, learners evaluate the scenario as they strategize for inquiries and proper administration. Active learning occurs when students have greater opportunities to engage with the case through interaction. This enables individuals to actively create knowledge instead of solely receiving it, thereby improving their abilities to collaborate and share information with their peers within a group setting [57,76]. Implementing CBL has been found to effectively cover a wide range of topics while establishing explicit learning objectives [13]. Additionally, CBL has been shown to enhance clinical knowledge, improve teamwork, foster the development of clinical skills, and encourage practice-based behavior [77,78].

While CBL has the potential to facilitate group discussions, it is imperative to implement appropriate measures for organizing the groups [13]. Learners and educators require additional time to prepare for each session adequately. This has a direct impact on the student’s schedule and their ability to prepare for exams. In addition, specific instructors’ demeanor, character, and temperament may result in a predominant influence over the educational process, leaving minimal opportunity for independent student investigation of the case, thereby impeding experiential learning.

Educators must receive adequate training to employ and value CBL use proficiently. According to Thistlethwaite et al., it is more efficacious to implement this learning technique through small group sessions with the participation of attentive learners and cases that are closely linked to clinical scenarios [79]. In addition, multiple-choice question assessments may be employed to evaluate and track students’ comprehension and cognitive approaches, thereby verifying that they proficiently employ this pedagogical approach [13].

Team-based learning

**Figure 2:**
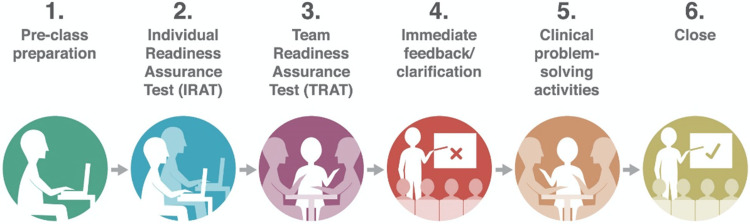
Steps in team-based learning. This figure was reproduced from Burgess et al. [82] under CC BY 4.0 (https://creativecommons.org/licenses/by/4.0/).

Team-based learning (TBL) has emerged as a prominent pedagogical approach in medical education, emphasizing student-centered learning [80]. TBL involves a pedagogical approach where a limited number of students work together in a group setting to apply educational concepts that are grounded in constructivist learning theory [13,81]. This approach involves various activities that require critical thinking, individually and as a team, including brainstorming and completing tasks. The instructor provides prompt feedback to the group after these activities. Utilizing a sequenced format offers prospects for the application and expansion of conceptual knowledge employing a series of steps encompassing preparation, readiness assurance testing, feedback, and the implementation of learning through clinical problem-solving tasks (Figure 2) [82].

TBL confers a significant benefit in enhancing communication skills and team-oriented strategies among student groups, which are crucial for effective patient care [83]. TBL has been implemented in various fields of dental education, including periodontics, oral medicine, oral radiology, removable prosthodontics, and fixed prosthodontics [57]. Research indicates that interactive teaching and learning methods have the potential to impart sustainable knowledge and improve student performance, resulting in greater satisfaction among students compared to traditional lecture-based classes [84].

TBL was initially developed by Larry Michaelson at Oklahoma University in the late 1970s. As the enrollment in his class increased from 40 to 120 students, he found it necessary to develop a new pedagogical approach that involved small-group learning in groups of 15 students [57]. The utilization of small-group discussions in a larger classroom setting, coupled with adequate preparation before class, is an effective means of facilitating peer-to-peer learning among students. A single instructor can facilitate all group activities within a larger classroom setting. The provision of compartmentalized space for group activities is deemed unnecessary as the groups can interact effectively within an open space [85]. TBL offers a significant benefit in enabling students to collaboratively identify solutions and make decisions, thereby promoting heightened motivation for learning, facilitating the creation of concept maps, and fostering deep learning [57]. According to Arja et al., implementing TBL through small group discussions led by instructors has resulted in higher student assessment scores than traditional didactic lectures [86].

Regrettably, empirical evidence indicates that a subset of learners and instructors encounter difficulties with this pedagogical approach, as some students do not appreciate the merits of collaborative work and perceive it as less effective and efficient than didactic learning [13]. Difficulties may arise in the transfer of knowledge from educators to learners. Both parties must have faith in the importance of implementing theoretical concepts through real-life scenarios.

Instructors who only partially adopt this learning approach and instead resort to imparting information to students risk impeding their creative process and critical thinking abilities [13]. This may result in students feeling dissatisfied and perceiving inadequate knowledge acquisition. To minimize potential disparities in knowledge among team members during group work, it is advisable to provide students with pre-recorded lecture notes and mandatory reading assignments before attending instructional sessions with the instructor. According to Burgess et al., using open-ended questions by instructors to encourage group discussions among students can lead to improved outcomes in student groups [80].

Competency-based education

There are multiple definitions of competency-based education (CBE). Nevertheless, there is consensus that outcome-based education (OBE) ensures the production of graduates with the knowledge, skills, and attitudes necessary to successfully serve their society and patients and meet the requirements of the national qualifications framework [87]. There is a tendency to refer to CBE and OBE interchangeably. In dentistry, however, CBE is frequently utilized rather than OBE [87].

CBE is a pedagogical approach that centers on the targeted performance attributes of healthcare practitioners. The CBE approach takes into account both societal and patient needs when determining the competencies and characteristics that a graduate should possess. These predetermined competencies and attributes serve as the basis for designing curriculum content, teaching and learning methods, assessment techniques, and educational settings and assistance [88]. CBE has been likened to OBE in various fields, particularly medicine. OBE is an educational methodology in which the curriculum is designed based on the learning outcomes that students must exhibit to advance and is compatible with cognitivism theory [89,90].

The controversial relationship between CBE and OBE is a subject of academic discourse. Specific individuals perceive CBE as a constituent element of the advancement of OBE [91]. Cate et al. argue that CBE takes a broader perspective than OBE by encompassing educational outcomes and considering OBE as a component of CBE [92]. From an alternative perspective, proficiency in CBE and achievements in OBE center on the professional level, where learners are anticipated to possess the capability to conduct independent practice and handle diverse professional predicaments [88].

CBE includes the following four characteristics: (a) learner goals concentrating on market demands, (b) modules organized in sequences, (c) students learning at their own pace, and (d) students being evaluated in authentic situations [11]. The primary objective of a CBE curriculum is the acquisition of competencies that are consistent with the profession and job market expectations of graduates. CBE in healthcare begins with a thoughtful evaluation of the competencies required of healthcare professionals to assist in meeting the healthcare needs of a specific country. In 1995, the American Dental Education and the Commission on Dental Accreditation (CODA) implemented CBE in the United States [11]. After two years, CODA designated it as the recommended method in their dentistry programs as it enhances student learning [93]. CODA 2008 reevaluated its pre-doctoral requirements and established CBE as the proposed curriculum for educating future general dentists [11].

The CBE-focused dentistry curriculum logically integrates all required competencies. It ensures that all student performances are supported by biomedical theory, clinical and social competencies, and physical skills. CBE learning objectives must be explicit, and evaluation must be flexible while highlighting several rules. It is imperative for dental students to exhibit proficiency achievement, gradual acquisition of knowledge, and the capacity to monitor their educational advancement [12,94,95].

Flipped classroom

**Figure 3:**
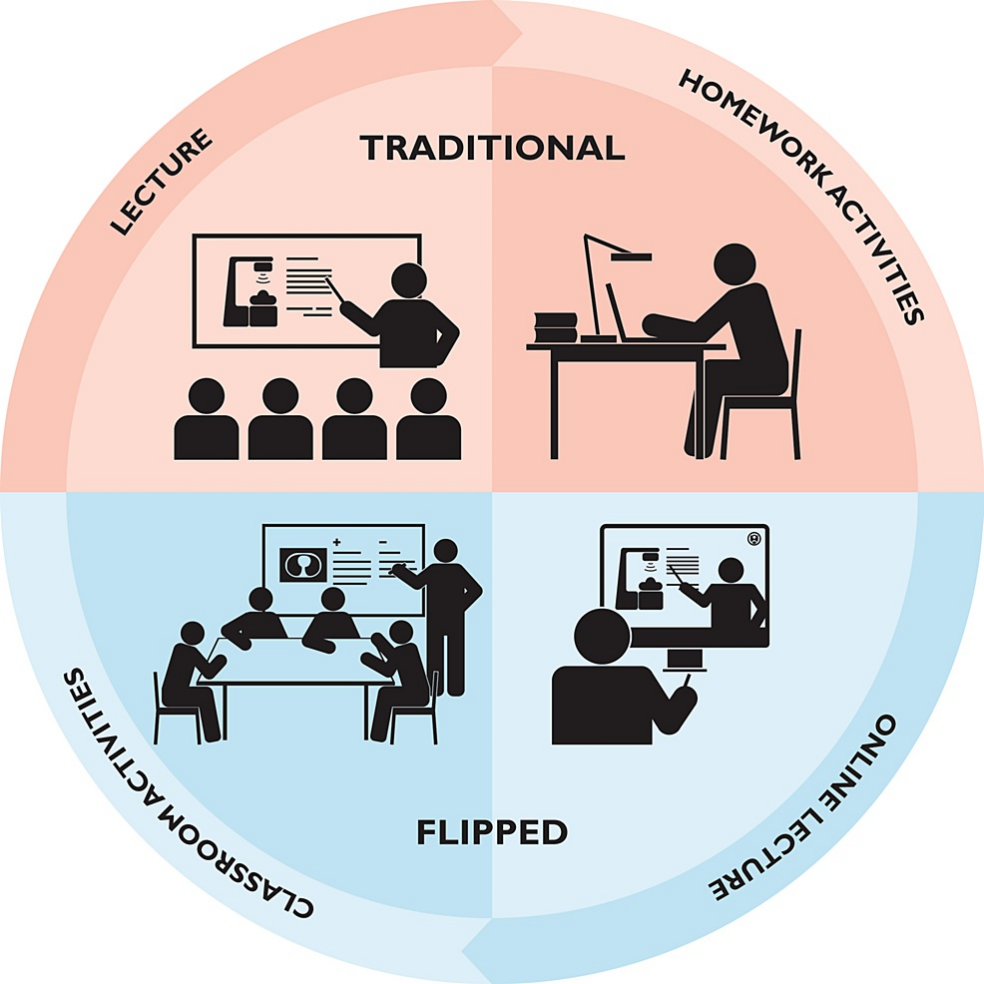
The difference between flipped learning and traditional learning. As shown in the top row, traditional education involves lectures and homework. The lower row shows flipped learning, which uses online lectures and classroom time for interactive tasks. The figure was reproduced from Kim et al. [99] under CC BY 4.0 (https://creativecommons.org/licenses/by/4.0/).

The flipped classroom is an innovative pedagogical approach that integrates blended learning methodologies by utilizing online, offline, or hybrid instructional materials outside of the conventional classroom environment and is supported by the constructivism theory [13,96]. The methodology emerged upon the publication of a YouTube video entitled “The Flipped Classroom” in 2010 [57,97]. It involves assigning didactic material that is traditionally covered in lectures to learners to be learned before class while utilizing classroom time for interactive and active learning, and a shift is observed from an instructor-centered approach to a self-directed learning paradigm (Figure 3) [13,98,99].

The flipped classroom model fosters self-directed learning by requiring students to seek supplementary resources to bolster the provided material. It is reportedly effective in educating dental students on various subjects, such as periodontal diagnosis, treatment planning, and child abuse in pediatric dentistry [57]. According to the literature, implementing the flipped classroom approach has been demonstrated to enhance students’ perception, learning outcomes, critical thinking abilities, and motivation compared to conventional lecturing techniques [13]. The integration of audio-visual aids offers students unrestricted access to educational resources, thereby promoting interactive and self-directed learning encounters [100].

Although flipped classrooms offer certain benefits, they have been criticized for their limited instructor involvement and inadequate opportunities for collaboration in clinical skills, which are crucial for effective clinical practice [13]. Additional obstacles involve recognized variations in cognitive capacities that learners may employ when studying from a digital display as opposed to printed materials, such as relatively substandard time management, susceptibility to distractions, and diminished aptitude for sustained focus [101].

The practical implementation of flipped classrooms necessitates the educator and learner’s active engagement. The demand for educators to meticulously plan and construct instructional tasks that promote student readiness and involvement is crucial. Incorporating strategies such as soliciting student feedback, fostering peer learning, and promoting pre-reading can facilitate the effective implementation of the flipped classroom model [13]. Various pedagogical approaches, such as administering a pre-class quiz, have been found to incentivize students to engage in pre-reading of specific subject matter, clarify uncertainties, and promote reflective learning [102].

Simulation-based learning

Simulation is a constructed representation of artificial illustration used to achieve educational objectives through experiential learning [13]. Simulation learning uses simulation aids to replicate and integrate real clinical scenarios into experiential theory [13,103]. Since the early days of medicine, simulation has been used haphazardly. Phantoms were created in the 16th century to teach obstetrical procedures and decrease maternal and infant mortality rates [104]. Today, it is common for students to practice their first injections on an orange and practice suturing on fabric [105].

However, modern medical technology application necessitates complex team interactions that call for improved training methods. Advanced simulators enable educators to alter student responses and reactions in ways that are impossible with actual patients by offering realistic representations of complex clinical environments [105]. While simulation-based learning (SBL) has the potential to replicate authentic clinical scenarios, it may serve as an initial practical encounter for students that necessitates the coordination of efforts, patience, cooperation, and helpful guidance [13]. Preparing and organizing a session can be a time-intensive task that requires adequate resources to ensure equitable opportunities for all students. The acquisition and upkeep of SBL equipment, including models, software, and facility areas, can entail significant costs and necessitate proper maintenance. Educators must receive sufficient training in the operation of all equipment.

The incorporation of simulation-based training in conjunction with conventional didactic lectures has demonstrated a reduction in errors and enhancement of medical procedure performance. Hence, it is recommended that simulation methodologies be employed in teaching intricate medical procedures to enhance the outcomes of patient care [13]. The level of student enthusiasm can be improved by reducing the size of groups and providing limited instructional guidance, which enables students to manage their tasks independently and promotes their participation in peer discussions.

Peer-assisted learning

Peer-assisted learning (PAL) involves the deliberate intention to assist others in achieving their learning objectives through explicit active helping and support among peers or matched companions, resulting in the development of knowledge and skill [106]. When there is an educational gap between students, it is often called near-peer learning [106]. The framework in question is a collaborative, analogical, and informal learning approach involving a cohort of enthusiastic individuals who support each other in pursuing knowledge [13]. PAL is based on Vygotsky’s social constructivism theory [107].

It has been observed that when students participate in active learning by teaching the material, they tend to analyze and restructure the information, resulting in a more profound comprehension and enhanced retention of the subject matter [108]. In addition, using this methodology helps to strengthen students’ knowledge, their confidence in speaking up, and their communication abilities. Simultaneously engaging in teaching and learning activities while offering feedback to peers has yielded cognitive and non-cognitive benefits for tutors [13].

Considerable efforts must be undertaken to carefully select tutors with exceptional proficiency to realize this learning approach’s potential fully. Perceived stigma related to peer knowledge, lack of motivation, and willingness to collaborate can constrain [13]. The successful implementation of PAL demonstrates the efficacy of peer learning, encouraging active student engagement across various levels. It is crucial to consistently provide students with training and practice sessions that offer direction and support [109].

It is also imperative for peer tutors to cultivate proficient communication abilities and ample self-assurance to guarantee the triumph of their endeavors [13]. The tutor and tutee’s progress monitoring can be achieved through evaluation, feedback, observations, and reflective logging [110]. If a desired outcome is not attained, educators can aid, adapt, or offer alternative pedagogical approaches to accommodate the academic requirements of their learners [13].

Observational learning

Observational learning is the process by which an observer tries to imitate a demonstrated behavior or movement [111]. Observational learning operates on the premise that the observer employs a cognitive representation acquired through observation to direct the subsequent execution of motor skills [111]. According to social learning theory, four subprocesses affect the acquisition of a motor skill when learning through observation, namely, attention, retention, reproduction, and motivation [111].

Observational learning entails the dedication of the motor system to acquiring knowledge, necessitating the implicit involvement of the observer [13]. Moreover, expeditious feedback creates a perception that it has an impact in the context of not only physical training but also observational acquisition.

Observational techniques are pivotal in acquiring proficiency in intricate medical procedures by augmenting learning and skill through observational practice. Enhancing the performance of medical procedures relies on the development of motor skill practice and comprehension of the fundamental mechanisms that govern these motor actions, which is imperative in creating more effective training systems [13]. Attention and retention play essential roles in the acquisition phase of motor skill acquisition [111]. If the ideas match with the execution, it is possible for the observer to recall their knowledge and experience accurately when recreating complex actions. Consequently, reproduction and motivation are advantageous for reproducing motor performance. It is challenging to detect discrepancies between cognitive representations and execution, so the observer must replicate the depicted motor abilities with enthusiasm and adeptness [111].

The integration of observational learning into the curriculum poses particular challenges. It is challenging for instructors to evaluate behavior as they need more control over students’ level of engagement in observing a specific technique or skill [13]. The establishment of observational sessions can be arduous and require significant physical effort. Similarly, it is noteworthy that the acquisition of motor skills through simulation or virtual training only encompasses a subset of the motor and sensory processes that are engaged in the complete process of motor skill development compared to the benefits derived from actual practice [13]. Students are exposed to visual stimuli that are related to the execution of movement. Nevertheless, they cannot access the neural connections associated with the motor periphery or afferent feedback. In addition, the demonstration may introduce observer bias, in which the students’ interest may influence their perception and interpretation of the demonstration.

Similarly, a temporary alteration in student conduct may occur, which is commonly called the “Hawthorne effect” [13]. The integration of diverse teaching methodologies can augment the learning process and foster the cultivation of clinical acumen. Hence, incorporating additional pedagogical approaches, such as observational learning, is crucial to mitigating partiality and addressing any deficiencies associated with a single instructional modality [112]. Observational learning encompasses physical demonstrations and dynamic visualization of processes through video or animations, which can also be beneficial [13].

Dental students are adults who enter professional school with various learning styles that have been honed over many years of study. Hence, employing diverse methodologies would aid in catering to the distinct requirements and competencies of the learners [113]. In addition, increasing students’ awareness of their preferred learning mode can improve their learning outcomes and assist them in actively meeting the academic expectations of dental school.

Contemporary methods of dental education are aimed at enhancing student engagement by incorporating active learning strategies and establishing connections between theoretical concepts and practical applications. Enhancing competency, promoting logical thinking, and fostering improved clinical reasoning are benefits of this practice. Contemporary learning methodologies offer individuals the liberty to delve into knowledge and facilitate chances for introspection within a regulated setting [13].

Motivation is a critical factor in a student’s disposition toward lifelong learning, and lifelong learners are distinguished by their motivation. The inherent attributes of an individual’s self-perception, aspirations, and anticipations can shape their drive to pursue knowledge. The ability to engage in continuous learning throughout one’s life is an inherent human trait that can be further enhanced by identifying one’s individual learning preferences [13]. According to Collins, it is crucial to encourage self-initiated learning methods due to their high persistence and pervasiveness [114]. The academic material should pertain to the tasks and responsibilities that hold significance to the students. It is recommended that facilitators clearly state the learning objectives and articulate how a given activity will enable learners to attain their desired outcomes [13].

Educators must have a comprehensive understanding of the individual requirements of their students and create instructional strategies that cater to those needs with a particular focus on fostering motivation. The establishment of social relationships, fulfillment of expectations, professional growth, and cognitive benefits are all factors that can contribute to creating a motivational atmosphere [13]. The ideal educational encounter entails an engaged and self-driven approach, which fosters student engagement, the exchange of ideas, and participation in discussion.

The prompt implementation and familiarity with diverse pedagogical approaches facilitate comprehension and support practical application in the clinical setting [13]. The presented learning approaches cater to the unique learning disparities among individuals and incorporate diverse learning tactics to enable students. Undoubtedly, the integration of contemporary pedagogical approaches will enhance the process of knowledge and skill acquisition [13].

It is also necessary to expand research efforts to encompass the analysis of effects on students and faculty [57]. Before implementing a methodology, institutions should ensure that they possess adequate infrastructure for the specific method. The smooth progression of the implemented innovation is of the utmost importance. Providing sufficient training for faculty and students before implementing any teaching methodology is imperative.

Additionally, it is essential to ascertain the efficacy of the implemented innovative techniques rather than solely focusing on their implementation. Continuous monitoring is necessary to determine the effectiveness of these methods in providing optimal benefits to students. Gathering feedback for both students and teachers is recommended [57]. The assessment of the system’s efficacy is crucial and serves as a means to identify and overcome any potential obstacles.

## Conclusions

Knowledge acquisition is a continuous and uninterrupted process, and it is crucial to acknowledge that learners exhibit diverse learning modalities. Contemporary pedagogical methods that prioritize the student as the focal point of the learning process utilize innovative and imaginative approaches to facilitate knowledge acquisition. This approach enhances professional proficiency by cultivating skills, expertise, and leadership within the field. Differentiated teaching models should be effectively and appropriately accommodated in dental education, beginning from the preclinical years. This necessitates flexibility in the educational approach.

It is recommended that dental educators consider their students’ knowledge and the philosophical underpinnings of dental education to enhance the prevailing pragmatic perspectives that are typically employed. This is achieved by providing a more comprehensive theoretical framework for dental education, ultimately leading to an improved student learning experience. The thought processes discussed can aid educators in restructuring curricula, instructional methodologies, learning goals, and assessment techniques.
